# Urinary incontinence during pregnancy: prevalence, experience of bother, beliefs, and help-seeking behavior

**DOI:** 10.1007/s00192-020-04566-0

**Published:** 2020-10-20

**Authors:** Heidi F. A. Moossdorff-Steinhauser, Bary C. M. Berghmans, Marc E. A. Spaanderman, Esther M. J. Bols

**Affiliations:** 1 Faculty of Health, Medicine and Life Sciences, Department of Epidemiology, Care and Public Health Research Institute (CAPHRI), Maastricht University, P.O. Box 616, 6200 MD Maastricht, The Netherlands; 2 Pelvic Care Unit Maastricht, CAPHRI, Maastricht University Medical Centre (MUMC+), Maastricht, The Netherlands; 3 Department of Obstetrics and Gynaecology, MUMC+, Maastricht, The Netherlands

**Keywords:** Help-seeking, Pelvic floor muscle exercises, Pre-partum, Prevalence, Quality of life, Urinary incontinence

## Abstract

**Introduction and hypothesis:**

Pregnancy and delivery are thought to induce urinary incontinence (UI), but its clinical impact is less known. Therefore, we investigated the prevalence of self-reported UI, level of experience of bother, and beliefs to gain a greater understanding of help-seeking behavior in adult pregnant women.

**Methods:**

A digital survey shared on social media was used for recruitment. The survey consists of: (1) demographic variables, (2) International Consultation on Incontinence Questionnaire-Urinary Incontinence Short Form (ICIQ-UI SF), (3) ICIQ Lower Urinary Tract Symptoms Quality of Life (ICIQ-LUTSqol), and (4) questions on beliefs and help-seeking behavior. For analysis, descriptive statistics and the independent samples t-test were used to determine differences between help- and non-help-seekers.

**Results:**

Four hundred seven women were eligible for data analysis. The prevalence of UI rises from 55.1% in the first to 70.1% in the third trimester, with an overall prevalence of 66.8%. Nearly 43.0% of the respondents reported UI occurring once a week or less; 92.5% of women lost a small amount; 90% reported slight to moderate impact on quality of life. Only 13.1% of the respondents sought help for their UI. The main reasons for not seeking help were: minimal bother and the idea that UI would resolve by itself. Help-seeking women showed significantly higher scores than non-help-seeking women regarding ICIQ-UI SF (*p* < 0.001), ICIQ-LUTSqol (*p* ≤ 0.001), and interference in daily life (*p* < 0.001).

**Conclusions:**

During pregnancy, UI affects two out of three women, but only one in eight women sought professional help. Non-help-seeking women experience less bother.

## Introduction

Urinary incontinence (UI) is the complaint of involuntary loss of urine [1]. The self-reported prevalence of UI in the antenatal period is widely researched. These prevalence numbers vary greatly throughout published reports (9–63%), depending on case definitions applied, recruited population, and study methodology. Pregnant women seem to differ regarding degree of experienced bother in relation to UI [2, 3]. Cautious interpretation of (high) prevalence rates is needed when case definitions used do not incorporate a measure of symptom bother as the crude UI prevalence rate may overestimate the prevalence rate of significant or bothersome UI. Therefore, the International Consultation on Incontinence (ICI) recommends prevalence numbers to be accompanied by a measure of bother [4].

For women with UI in the general population, it is known that bothersome UI, but also urgency UI (UUI), and UI severity (defined by the ICI as frequency of UI times volume of UI) are associated with help-seeking behavior [4–6]. Although pregnancy is known for its provoking effect on UI, knowledge on experience of UI bother and help-seeking behavior in this period is lacking. Furthermore, it is unclear which specific bothersome factors and beliefs are the main contributors to help-seeking behavior. Guidelines on UI in women in general recommend pelvic floor muscle training (PFMT) as a first-line treatment option [7, 8].

To inform health care providers, researchers, and policy makers, it is important to have accurate prevalence rates as well as knowledge on pregnant women’s beliefs and help- seeking behavior. Therefore, we aim to investigate the prevalence of self-reported UI, level of experience of bother, and beliefs to explain help-seeking behavior in pregnant women in The Netherlands.

## Materials and methods

### Study design

A cross-sectional design was used to describe the prevalence, bother, beliefs, and help-seeking behavior of pregnant women. The Medical Ethics Committee of the Maastricht University Medical Centre (MUMC+) was consulted. It was stated that ethical approval was not necessary because of the non-invasive character of the study (MECC 019–1320). Pregnant women ≥ 18 years old, regardless of parity and weeks of gestation, and able to fill in a digital survey, were eligible to participate. Based on an overall expected prevalence of UI of 41%, a Z statistic of 1.96, and precision of 0.05, a minimal sample size of 371 women was estimated to fill in the survey [9]. Nationwide midwifery and pelvic physiotherapy practices were among others asked to share a social media message (using Facebook and LinkedIn) containing brief information on the study (goal, eligibility) and a link to the patient information letter and digital survey. Before proceeding to the anonymized digital survey, eligible women signed informed consent forms electronically, in agreement with ethical regulations. The survey took 10 to 15 min to complete.

### Outcome measures

The survey consisted of four parts: (1) demographic variables such as age, trimester of pregnancy, educational level, and parity, (2) International Consultation on Incontinence Questionnaire-Urinary Incontinence Short Form (ICIQ-UI SF) [10], (3) International Consultation on Incontinence Questionnaire Lower Urinary Tract Symptoms Quality of Life (ICIQ-LUTSqol) [11], and (4) questions on beliefs and help-seeking behavior regarding UI.

The ICIQ-UI SF provides an indication of UI severity and consists of four questions. The first question assesses frequency of UI, with a score of 0 (never losing urine) to 5 (losing urine all the time). The second question describes the amount of urine loss, with four response categories ranging from 0 (no loss) to 6 (large amount). The third question assesses the impact of UI on daily life, ranging from 0 (not at all) to 10 (a great deal). The total score ranges from 0 (no impact of UI on quality of life) to 21 (very severe problem). The total score is divided into four severity categories: slight (1–5), moderate (6–12), severe (13–18), and very severe (19–21) [12]. A fourth question on the occurrence of symptoms of UI was used to indicate SUI or MUI [13]. A respondent was considered to have SUI when leaking urine with a cough or a sneeze and/or when physically active/exercising, but not before getting to the toilet. UUI is considered when the respondent leaks, because of irresistible need to void, before getting to the toilet. A respondent with MUI experiences both SUI and UUI.

The ICIQ-LUTSqol is a condition-specific health-related quality of life questionnaire (20 questions) adapted for use within the ICIQ structure from the King’s Health Questionnaire [11]. It contains 19 questions that can be scored on life restrictions, emotional aspects, and preventive measures. It is scored on a four-point Likert scale ranging from 1 (not at all) to 4 (a lot). Three questions on relationships, sex life, and family life included additionally ‘not applicable.’ ‘Not applicable’ was considered as not affecting daily life [14]. The sum score ranges between 19 and 76. A higher score indicates a higher impact on quality of life. Every question is accompanied by a question regarding experienced bother [ranging from 0 (no bother) to 10 (extreme bother)]. It is arbitrarily decided that a score of at least 5 indicates significant bother on a specific item. The 20th question is on how much urinary symptoms interfere with daily life. This is scored between 0 to 10 (similar to experienced bother). Both the ICIQ-UI SF and ICIQ-LUTSqol are rated as ‘high-quality’ questionnaires and are recommended by the ICI [4]. The ICIQ-UI SF and the ICIQ-LUTSqol were provided in the Dutch language by the Bristol Urological Institute [15].

All respondents at least filled in the demographic variables and ICIQ-UI SF. Answering ‘never losing urine’ at the frequency item of the ICIQ-UI SF indicated continence and consequently the survey was finished. When reporting UI, women completed the remaining two parts on quality of life and help-seeking behavior.

The questions on beliefs and help-seeking behavior were self-constructed. Selection of question and answer options was based on models explaining help-seeking behavior and discussion with experts in the field (epidemiologists and obstetrician/gynecologist) and modified accordingly [16, 17]. Moreover, questions were reviewed by an expert for readability and comprehensiveness, followed by field testing. Ultimately, six questions were developed including four topics on health-seeking behavior [actual help-seeking, reason(s) to (not) seek help, reason to seek help in the future, and consulted health care provider(s)] and two topics on beliefs (self-perceived prognosis and self-perceived best intervention to treat UI in general).

### Data analysis

Data were analyzed using descriptive statistics presented as proportions [frequency and means (SD)]. An independent sample t-test was conducted to compare help-seekers and non-help-seekers regarding UI severity (ICIQ-UI-SF total score), bother (ICIQ-LUTSqol total score), and interference in daily life. A chi-square test was used to test relationships between categorical variables. The effect size is estimated with Cohen’s *d*. Cohen’s *d* presents the difference between groups (help-seekers and non-help-seekers) in standard deviation units. To interpret the strength of the effect size, we follow the guidelines proposed by Cohen: 0.2 = small, 0.5 = medium, and 0.8 = large. An alpha of 0.05 is considered significant. Analyses were done using IBM Statistical Package for Social Sciences (SPSS), version 26.0 (New York, NY, USA).

## Results

In March and April 2020, 415 women filled in the survey. Eight women did not complete the survey after giving consent and were excluded from analysis. This left 407 women eligible for data analysis. The mean age was 30.4 years (SD 3.9, range 18–49), of which 146 (35.9%) were nulliparous (Table 1). The prevalence of UI rose from 55.1% (27/49) in the first trimester to 70.1% (162/231) in the third trimester. The overall prevalence of UI was 66.8% [272/407, 95% confidence interval (CI) (62.3–71.3)]. SUI [76.8% (209/272)] was the most frequently reported type of UI. Nulliparous women reported a significantly lower overall prevalence of 47.9% (70/146) compared with 77.4% (202/261) for (multi)parous women (*p* < 0.001).

**Table 1:** Background variables and urinary incontinence prevalence

Background variables (*N* = 407)			*N* (%)
Age (mean, SD, range)	30.4 (3.9, 18–49)		
Education	Primary education		2 (0.5)
	Secondary education		185 (45.5)
	Tertiary education		220 (54.1)
Parity	Nulliparous		146 (35.9)
	Multiparous		261 (64.1)
Pre-partum period	Trimester 1 (1–13 weeks)		49 (12.0)
	Trimester 2 (14–26 weeks)		127 (31.2)
	Trimester 3 (27–42 weeks)		231 (56.8)
UI prevalence (by)	Overall		272 (66.8) 95% CI (62.3–71.3)
	Type	SUI	209 (76.8)
		UUI	11 (4.0)
		MUI	34 (12.5)
		Other (such as: UI during sleep or UI for no obvious reason)	18 (6.6)
	Trimester	1st (1–13 weeks)	27/49 (55.1) 95% CI (41.2–69.0)
		2nd (14–26 weeks)	83/127 (65.4) 95% CI (57.1–73.7)
		3rd (27–42 weeks)	162/231 (70.1) 95% CI (64.2–76.0)
	Parity	Nulliparous	70/146 (47.9)
		Primi-/multiparous	202/261 (77.4)

N = number, % = percentage, SD = standard deviation, CI = confidence interval, UI = urinary incontinence, SUI = stress urinary incontinence, UUI = urgency urinary incontinence, MUI = mixed urinary incontinence

Nearly 43.0% (116/271) of the respondents reported UI frequency of once a week or less, and in 91.1% (247/271) of cases it was a small amount of urine per episode (Table 2). Ninety percent of the women reported slight (33.7%, 91/270) to moderate (56.3%, 152/270) impact of UI based on the ICIQ-UI SF score, whereas the mean ICIQ-LUTSqol total score was 28.2 (SD 7.2, range 19–57). The mean interference in daily life based on ICIQ-UI SF was 3.0 (SD 2.7, range 0–10), whereas 29.9% (81/272) of women indicated a significant interference of ≥ 5. The ICIQ-UI SF and ICIQ-LUTSqol total scores and interference in daily life did not increase by trimester. Pregnant women experienced significant bother in relation to having UI on only 2 out of 19 questions on the ICIQ-LUTSqol, namely ‘changing of wet underclothes’ and ‘worry because of smell.’

**Table 2:** ICIQ-UI SF questionnaire results

ICIQ-UI-SF		*N* (%)
ICIQ Frequency	About once a week or less often	116 (42.6)
	Two or three times a week	53 (19.6)
	About once a day	36 (13.3)
	Several times a day	63 (23.3)
	All the time	3 (1.1)
ICIQ Amount	None	4 (1.5)
	A small amount	247 (92.5)
	A moderate amount	20 (7.5)
	A large amount	0 (0.0)
ICIQ-UI SF overall interference (range 0–10)	≥5	81 (29.9)
ICIQ-UI SF total score mean (SD, range)	0–21	7.5 (3.6, 0–19)
Categories ICIQ-UI SF 2 missing	Slight (1–5)	91 (33.7)
	Moderate (6–12)	152 (56.3)
	Severe (13–18)	26 (9.6)
	Very severe (19–21)	1 (0.4)

ICIQ-UI SF = International Consultation on Incontinence Questionnaire Urinary Incontinence Short Form, N = number, % = percentage, SD = standard deviation

**Fig. 1:**
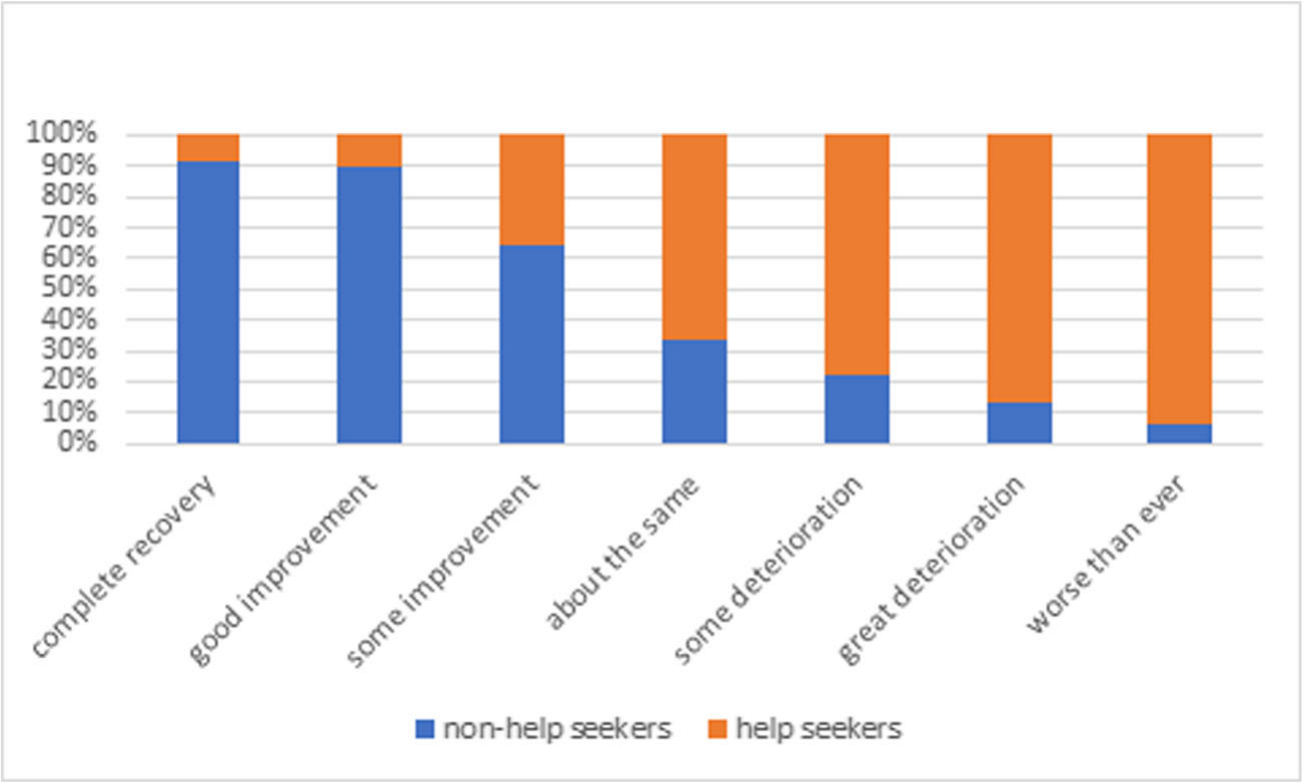
Beliefs about prognosis of urinary incontinence if help is not sought among non-help-seekers and help-seekers

In total, 13.1% (35/267) of the respondents with UI sought help (Table 3). The majority of women seeking help (91%, 32/35) visited a (specialized) physiotherapist. Seven women (21.9%) reported that they initially visited the pelvic physiotherapist for another health problem, such as pelvic girdle pain. The reasons provided for not seeking help were: minimal bother (53%, 123/232), the idea that UI would improve by itself (38%, 89/232), and wanting to postpone until after the delivery (32%, 75/232). The most important reasons for seeking help in the future were: the constant use of pads (47%, 110/232), the feeling that others can smell the urine loss (33%, 77/232), and leaking/getting wet clothes (30%, 70/232). Fifty-six percent (130/232) of women who did not seek help in contrast to 5.8% (2/35) of the women who did seek help for their UI thought that their UI would completely resolve or improve a great deal in the future. Figure 1 shows the beliefs about prognosis of UI among non-help-seeking and help-seeking women as relative percentages of 100%. Of all women with UI, 71.5% (191/267) thought that the best way to treat their UI would be pelvic floor muscle exercises.

**Table 3:** Beliefs and help-seeking behavior in relation to urinary incontinence

Beliefs		
Prognosis UI without seeking help	Help-seekers (*N* = 35)	Non-help-seekers (*N* = 232)
Complete recovery	1 (2.9)	71 (30.6)
Good improvement	1 (2.9)	59 (25.4)
Some improvement	3 (8.6)	36 (15.5)
About the same	13 (37.1)	44 (19.0)
Some deterioration	7 (20.0)	13 (5.6)
Great deterioration	8 (22.9)	8 (3.4)
Worse than ever	2 (5.7)	1 (0.4)
Best way to solve UI		
Surgery	3 (8.6)	3 (1.3)
Medication	0 (0)	0 (0)
Pelvic floor muscle exercises	24 (68.6)	167 (72.0)
It will resolve by itself	0 (0)	30 (12.9)
There is no solution	0 (0)	3 (1.3)
I do not know	5 (14.3)	22 (9.5)
Other	3 (8.6)	7 (3.0)
Help-seeking	Help-seekers	Non-help-seekers
Reason to seek help	I sought help because^*^	I will seek help in the future if^#^
Getting wet clothes/leaking through	6 (17.1)	70 (30.2)
Need to use pad all the time	7 (20.0)	110 (47.4)
Others can smell me	0 (0)	77 (33.2)
Hindrance during sports	5 (14.3)	29 (12.5)
Hindrance during work	3 (8.6)	56 (24.1)
Hindrance playing with children	0 (0)	41 (17.7)
Hindrance during household tasks/activities	1 (2.9)	27 (11.6)
I do not know	0 (0)	28 (12.1)
Other reason(s)	13 (37.1)	30 (12.9)
Reason not to seek help		Non-help-seekers (N = 232)
Minimal bother		123 (53.0)
It will improve by itself		89 (38.4)
Postpone until after delivery		75 (32.3)
Lack of time		8 (3.4)
No childcare		5 (2.2)
Costs		2 (0.9)
No transport		0 (0.0)
Other		22 (9.5)

N = number, UI = urinary incontinence, ^*^ = one answer possible, ^#^ = multiple answers possible

Help-seeking women showed significantly higher scores than non-help-seeking women regarding ICIQ-UI SF (*p* < 0.001), ICIQ-LUTSqol (p < 0.001), and interference in daily life (p < 0.001), with corresponding large effect sizes (ICIQ-UI SF total score: Cohen’s *d* = 0.80, ICIQ-LUTSqol total score: Cohen’s *d* = 0.74, and interference in daily life Cohen’s *d* = 0.76).

## Discussion

### Principal findings

This study showed that the crude prevalence of self-reported UI during pregnancy is high (66.8%) and rises by trimester. SUI is the most frequently reported type of UI (76.8%) with a notable difference between nulliparous (47.9%) and parous women (77.4%) in overall UI prevalence. The severity of UI is slight (33.7%) to moderate (56.3%); total bother is experienced as low, and only less than one third of women indicate a significant impact in daily life. Only the presence of the factors ‘changing of wet underclothes’ and ‘worry because of smell’ were considered a significant bother. Only 13% of respondents sought help for UI. The responders who sought help were often already seeing a (specialized) physiotherapist for other pregnancy-related problems, such as pelvic girdle pain. The pelvic floor muscles are reported to play an important role in trunk stability [18]. Therefore, it is common practice for (specialized) physiotherapists to discuss any incontinence with pregnant women presenting with pelvic girdle pain. This encourages the women to mention their UI and seek help [19]. To our knowledge, this is the first study reporting on the percentage of women who actually seek help for their UI during pregnancy. However, the numbers on help-seeking might have been influenced by the fact that social media messages were sent by both midwifery and pelvic physiotherapy practices. The respondents who did not seek help stated that their UI did not bother them a lot (53%).

Several factors might explain why pregnant women with UI do not seek help. First, nearly 40% of the respondents thought that UI would improve spontaneously after delivery. However, pregnant women might be insufficiently aware that women with UI during pregnancy have a two- to six-fold risk of UI post-partum, depending on the severity of UI in pregnancy and the post-partum period [20]. Second, the reported overall bother was low, and impact on quality of life due to UI was not greatly affected. A higher level of bother is associated with help-seeking [19, 21]. Third, only 4% of the respondents had UUI, and especially women with UUI are reported to have lower quality of life than women with SUI and seek more help [5]. Fourth, 32% of the respondents wanted to wait until after the delivery to seek help. In contrast to the non-help-seekers (28.4%), most of the help-seekers (85.7%) thought that without help their UI would remain the same or deteriorate post-partum. This is consistent with Schreiber et al. who reported that women who are afraid that their UI will get worse are triggered to seek help [22].

Over 70% of all respondents reported that they think that pelvic floor exercises are the best treatment option for UI. This does not mean that these women actually exercise. Burgio et al. found that although 84.6% of women had heard of pelvic floor muscle exercises, only 46.7% of the women really did exercise during pregnancy [20]. Women want to be informed about pelvic floor dysfunctions preferably during pregnancy [19, 21]. Antenatal classes may be a perfect opportunity to discuss pelvic floor-related issues and misconceptions like the fact that UI will resolve by itself. If the importance and positive effect of PFMT are explained, women may be more willing to do their exercises [23]. Women who attend or have attended antenatal classes are more likely to practice pelvic floor muscle exercises than women who have not [24]. Another option to inform women might be with a mobile app (mApp). However, at the moment the only existing evidence-based mApp is not specifically developed for pregnant women and focusses on self-treatment and adherence to UI treatment and not on providing information on pelvic floor dysfunctions in pregnancy [25]. Although PFMT is an effective and well-established treatment option for women with UI, the treatment effect for UI during pregnancy is still uncertain [26]. Heterogeneity in studies due to differences in characteristics such as parity, PFMT programs, and control interventions may underlie the absence of robust evidence of effectiveness. Therefore, studies compensating for this heterogeneity are still needed to investigate the direct or remote effect of PFMT on UI during pregnancy.

Screening for the presence of UI and the degree of bother it causes in daily life (e.g., on activity and participation level) by health care professionals who see pregnant women is relevant to check for misconceptions and to have proper indications for subsequent interventions. However, health care professionals report not having enough time and knowledge to discuss UI [27].

### Clinical and research implications

The difference between the crude prevalence of UI and bothersome prevalence of UI during pregnancy demonstrates clearly the importance of reporting both prevalence numbers and the experience of bother in relation to UI [4]. This study reveals large effect sizes between help- and non-help-seekers regarding ICIQ-UI SF total, ICIQ-LUTSqol total scores, and interference in daily life. This indicates that non-help-seeking pregnant women experience little bother, just like women in the general population [21]. This is an important factor to consider in care planning and research as less bothered women will be not known to the health care system.

### Strengths and limitations

A strength of this study is the large nationwide sample. Another strength is the use of high-quality and recommended questionnaires to measure the prevalence and bother of UI and impact on quality of life. To our knowledge, this is the first study to use the ICIQ-LUTSqol to study bother extensively in pregnant women.

This survey has several limitations. First, women in The Netherlands who do not speak Dutch could not fill in the survey. This might have influenced the outcome regarding the knowledge on the best treatment option for UI. Non-native speakers are less likely to be familiar with possible treatments, e.g., pelvic floor muscle exercises [24]. Second, we did not ask if UI occurred before the first pregnancy or in previous pregnancies. Therefore, we do not know at what stage in their obstetric history pregnant women experienced new onset UI. The third limitation comprises the possible risk of bias due to the accessibility of social media for recruitment. Finally, the non-response rate is not known. However, we do know that the average age and education level are comparable to those in another large study performed in pregnant women in The Netherlands [28].

## Conclusion

UI is highly prevalent throughout pregnancy with prevalence increasing by trimester. However, the majority of women were only slightly bothered by their UI and relatively few women sought help.
